# Designing for medication adherence in inflammatory bowel disease: multi-disciplinary approaches for self-administrable biotherapeutics

**DOI:** 10.1016/j.eclinm.2024.102850

**Published:** 2024-10-10

**Authors:** Vivian Rachel Feig, Sufeng Zhang, Ashka Patel, Bruna Santos, Ziliang Kang, Sharmeel Wasan, Ana Beloqui, Giovanni Traverso

**Affiliations:** a Division of Gastroenterology, Department of Medicine, Brigham and Women's Hospital, Harvard Medical School, Boston, MA, USA; b Koch Institute for Integrative Cancer Research, Massachusetts Institute of Technology, Cambridge, MA, USA; c Department of Mechanical Engineering, Stanford University, Stanford, CA, USA; d Department of Biomedical Engineering, Stony Brook University, Stony Brook, NY, USA; e Department of Bioengineering, Northeastern University, Boston, MA, USA; f Department of Mechanical Engineering, Massachusetts Institute of Technology, Boston, MA, USA; g Department of Gastroenterology, Boston Medical Center, Boston, MA, USA; h Advanced Drug Delivery and Biomaterials, Louvain Drug Research Institute, Université catholique de Louvain, Brussels, Belgium; i WEL Research Institute, Wavre, Belgium

**Keywords:** Inflammatory bowel disease, Biotherapeutics, Drug delivery, Medication adherence

## Abstract

Biotherapeutics are among the therapeutics that have revolutionized standard inflammatory bowel disease (IBD) treatment, which was previously limited to mesalamine, 5-aminosalicylic acid, corticosteroids, and classical immunosuppressants. Self-administrable biotherapeutics for IBD would enable home-based treatment and reduce the burden on medical infrastructure. Self-administration is made possible through subcutaneous injectable, oral, and rectal dosage forms. Nevertheless, the full benefits of self-administration cannot be realized without first addressing the issue of medication adherence, which remains woefully inadequate for IBD biotherapies. Some of the major barriers to medication adherence in IBD are the route of administration, frequency of administration, and undesired side effects. In this review, we identify the main physiological and engineering constraints that underlie these three barriers to adherence. We then highlight key technological and behavioral innovations—spanning multiple scientific disciplines—that can be leveraged to design novel therapies and interventions that improve adherence to self-administered IBD biotherapies.

**Search strategy and selection criteria**

References for this Review were identified through searches of Google Scholar and PubMed using the individual or combinations of the following search terms: “inflammatory bowel disease”, “biotherapeutics”, “medical adherence in IBD”, “injectable biotherapeutics”, “subcutaneous”, “intravenous infusion”, “oral therapies”, “rectal administration”, “behavioral interventions”, “monitoring technologies”, “drug delivery”, “formulations”, and “devices” for publications between January 1, 2000 and March 2024, without imposing language restrictions. For selection, when possible, we prioritized more recent articles and articles that were specific to inflammatory bowel disease (IBD) biotherapies. However, we did not exclude relevant older publications or broader engineering-focused papers, as long as their content could be applicable to IBD biotherapeutics.

## Introduction

Inflammatory bowel disease (IBD), which encompasses ulcerative colitis (UC) and Crohn's disease (CD), is a chronic illness which, if left untreated, leads to significant morbidity and disability.^1^^,^^2^ Disease management commonly comprises immunosuppressive therapy through biologic drugs and small molecules aimed at reducing inflammation. Remarkable advances in drug development have been achieved in IBD treatment, including anti-tumor necrosis factor (anti-TNF) drugs (infliximab, adalimumab, certolizumab, and golimumab), anti-integrins (vedolizumab and natalizumab), and antagonists to interleukin (IL)-12 and IL-23, comprising anti-IL-12/23 antibody (ustekinumab), anti IL-23A antibody (risinkizumab), and IL-23 p19 antagonist (mirikizumab).^3^ Medications with these biotherapeutics are available or being developed via various administration routes, including intravenous (IV) injection, subcutaneous (SC) injection, and oral/rectal routes.

Among the biotherapeutic options for IBD, self-administration is a major driver for patient preference,^3^ particularly since IBD has a relapsing-remitting course that requires life-long medication and care.^4^ Moreover, self-administration reduces costs and hospital resource expenditure, the importance of which has only become more apparent since the recent COVID-19 pandemic.^5^ While self-administrable oral and rectal formulations are also being developed,^6^^,^^7^ SC injection remains the most clinically utilized non-IV format for biologic drugs (Table 1): In 2020, SC formulations of two IV biologic drugs (infliximab and vedolizumab) were made available to some patients,^8^ with vedolizumab SC made available in the US in 2023.^9^ Both SC formulations demonstrated pharmacokinetics, efficacy, safety, and immunogenicity profiles comparable to IV.^8^ This leads to improvement in quality of life, reduction of time needed to travel to the hospitals, and consequently reduces costs for the patient.^3^^,^^5^^,^^8^ Indeed, the pipeline for upcoming IBD monoclonal antibodies consists entirely of therapies in which long-term maintenance is achieved using recurring SC injections, after an induction period using either SC or IV.^8^

**Table 1:** Commercially available biologics for treatment of IBD.

Molecule	Brand name	Route of administration	Volume/concentration	Induction dosing	Maintenance dosing
**Anti-TNFα**					
Infliximab	Remicade (Janssen)	IV	250 ml, 0.4–4 mg/ml	Wk 0, 2, 6: IV inf (5 mg/kg)	5 mg/kg IV inf every 8 wks
Infliximab (Biosimilar)	Inflectra (Celltrion/Pfizer)	IV	250 ml, 0.4–4 mg/ml	Wk 0, 2, 6: IV inf (5 mg/kg)	5 mg/kg IV inf every 8 wks
Infliximab (Biosimilar)	Zymfentra (Celltrion)	SC (pre-filled pen or pre-filled syringe)	1 ml, 120 mg/ml	N/A – maintenance treatment only	1 × 120 mg SC inj every 2 wks
Adalimumab	Humira (Abbvie)	SC (pre-filled pen)	0.4 or 0.8 ml, 100 mg/ml	Wk 0: 2 × 80 mg inj Wk 2: 1 × 80 mg inj	1 × 40 mg SC inj every 2 wks
Certolizumab	Cimzia (UBC)	SC (pre-filled syringe)	1 ml, 200 mg/ml	Wk 0, 2, 4: 2 × 200 mg inj	2 × 200 mg SC inj every 4 wks
Golimumab	Simponi (Janssen)	SC (autoinjector or pre-filled syringe)	1 ml, 100 mg/ml	Wk 0: 2 × 100 mg inj Wk 2: 1 × 100 mg inj	1 × 100 mg SC inj every 4 wks
**Anti-Integrins**					
Vedolizumab	Entyvio (Takeda)	IV, SC (pre-filled syringe/pen)	IV: 250 ml, 1.2 mg/ml SC: 0.68 ml, 159 mg/ml	Wk 0, 2, 6: IV inf. (300 mg)	300 mg IV inf every 8 wks or 1 × 108 mg SC inj every 2 wks
Natalizumab	Tysarbi (Biogen)	IV	100 mL, 3 mg/ml	Wk 0: IV inf. (300 mg)	300 mg IV inf every 4 wks
**Anti interleukin (IL)-12 and IL-23 antagonists**					
Ustekinumab	Stelara (Janssen)	IV, SC (pre-filled syringe)	IV: 250 ml, 0.5–2 mg/ml SC: 1 ml, 90 mg/ml	Wk 0: IV inf. (260–520 mg)	1 × 90 mg SC inj every 8 wks
Mirikizumab	Omvoh (Eli Lilly)	IV, SC (pre-filled pen)	IV: 15 ml, 20 mg/ml SC: 1 ml, 100 mg/ml	Wk 0, 4, 8: IV inf. (300 mg)	2 × 100 mg SC inj every 4 wks
Risinkizumab	Skyrizi (AbbVie)	IV, SC (pre-filled pen)	IV: 10 or 20 ml, 60 mg/ml SC: 1.2 or 2.4 ml, 150 mg/ml	Wk 0, 4, 8: IV inf. (600 mg for CD and 1200 mg for UC)	1 x 180 mg or 1 x 360 mg SC inj every 8 wks

Abbreviations: CD, Crohn's disease; inf, infusion; inj, injection; IV, intravenous; SC, subcutaneous; UC, ulcerative colitis; wk, week.

Despite the importance of IBD biotherapeutics, patient non-adherence remains a major issue.^10^ Poor adherence to biologic therapy not only leads to poor symptom management and increasing risk of disease flare, but can also cause patients to develop auto-antibodies that make subsequent treatment less effective.^10^ In the largest study conducted on non-adherence to IBD biologic drugs, sub-optimal adherence to the drug adalimumab (an SC injection) below a threshold of 86% led to an increased risk of hospitalization and likelihood of necessitating corticosteroids. Shockingly, more than 20% of patients in the study fell below this threshold.^11^

There is, therefore, an urgent need to identify approaches to improve patient adherence to IBD biologics. In this review, we highlight the major barriers to adherence that have been identified from IBD patient studies. We then identify key technological approaches being explored by the broader drug delivery community that we argue can be effective in improving adherence to IBD biotherapeutics, as well as behavioral interventions like education. Finally, we conclude with an outlook on additional commercial and regulatory considerations for these approaches to become feasible within the IBD therapeutic landscape.

## Major barriers to adherence to IBD biotherapeutics

There have been numerous attempts to study the IBD patient population to try and identify major risk factors for poor adherence (Table 2). This is a surprisingly difficult task, as it is non-trivial to even obtain reliable data on adherence rates. For instance, medication possession ratio (MPR) is a commonly used metric, but is one that only tracks the rate at which prescriptions are filled,^10^ not actual administration of medication. On the other hand, clinical questionnaires^19^ that rely on self-reporting are subject to bias and reporting error, though are also more likely to directly reveal the underlying rationales for non-adherence.

**Table 2:** Summary of medication adherence studies.

Cohort size	Cohort demographics	Types of IBD drugs included	Adherence metric	Risk factors identified	Date	Reference
460	85% UC, 15% CD; mean age 37 years; 92% Caucasian	Adalimumab, certolizumab, golimumab, ustekinumab (all self-injectable SC)	MPR, non-adherence: MPR<86%	CD diagnosis, current narcotic use, psychiatric history, prior biologic use, smoking, Medicaid insurance	2020	10
365	18% UC or IC, 82% CD; mean age 41 years; 60% female; 87% Caucasian	Vedolizumab, infliximab, adalimumab, and certolizumab pegol	Modified MPR (mMPR), non-adherence: mMPR<100%	Self-administered biologics (as opposed to clinician-administered), younger age, noncommercial insurance	2018	12
1663	63% CD, 35% UC, 2% IC; mean age 44 years; 64% female; 87.2% members of the French IBD association (ADA)	IV infliximab, SC adalimumab	Mailed questionnaire	Younger age, smoking, constraints related to treatment, anxiety, moodiness	2011	13
106	IBD; median age 32 years; 48% female; UK based	IV infliximab, SC adalimumab	Medication Adherence Report Scale (MARS) Data from medical claims and outpatient specialist visits	Self-reported reasons for non-adherence: for infliximab, inconvenience; for adalimumab, forgetting.	2011	14
108	Only CD; median age 35 years; 64.8% female; French university based.	SC adalimumab	Missed/delayed doses Data from systematic questioning at outpatient specialist visits	40 mg dose biweekly (as opposed to 80 mg biweekly). Self reported reasons for non-adherence: forgetfulness, infection, travel, intentional nonadherence, pharmaceutical supply issues, side effects, pregnancy, and CD-related hospitalization.	2011	15
86079	CD, RA, PA, AS, JIA, CPP; mean age 52; 67% women, 81.2% Caucasian	SC adalimumab	MRA, data from pharmacy refill data	Retail Pharmacy (versus a Specialty Pharmacy), reimbursement by federal programs, female, person of color, Hispanic/Black (versus White)	2010	16
274	Only CD; mean age 33 years; 58% female; US university based	IV infliximab	Number of no-shows Data from administrative data, pharmacy refill data, registry	Female gender, Medicaid insurance, maintenance Dosing (>18 weeks since induction)	2006	17
571	56% UC, 38% CD, 6% IC; Pediatric patients, mean age 13; 45% female; Canada based	IV infliximab, oral small molecule drugs, enemas, vitamins, herbal supplements	Adherence: taking >80% of prescribed doses Data from mailed questionnaire and IBD patient database	Older age (14.6 years versus 13.0 years), longer disease duration, reported use of herbal medications. Self-reported reasons for non-adherence: forgetfulness, feeling better and too many medications.	2000	18

Abbreviations: AS, ankylosing spondylitis; CD, Crohn's disease; CPP, chronic plaque psoriasis; IC, indeterminate colitis; IV, intravenous; IBD, inflammatory bowel disease; JIA, juvenile idiopathic arthritis; MARS, Medication Adherence Report Scale; mMPR, modified medication possession ratio; MPR, medication possession ratio; MRA, medication refill adherence; PA, psoriatic arthritis; RA, rheumatoid arthritis; SC, subcutaneous; UC, ulcerative colitis.

Moreover, cohort characteristics vary significantly between studies, ranging from small single-center studies of less than 100 people, to larger studies with over 1000 participants. The studies we found also overwhelmingly focused on patients whose average age was around 40 and who were in Western, developed countries.^10^^,^^12^, ^13^, ^14^, ^15^, ^16^, ^17^, ^18^ Clearly, more studies are needed to obtain a more comprehensive picture of patient preferences and habits. Intriguingly, some studies identified correlations between poor adherence and identification with certain demographics. For instance, risk factors for poor adherence to injectable biotherapies include female sex,^20^ anxiety,^20^ prior narcotic use,^10^ and history of psychiatric disease.^10^ Children and adolescent patients also tended to have poorer adherence to IBD therapies compared with adults.^21^ These findings warrant further investigation, as they suggest an opportunity for human-centered design to specifically focus on tailoring therapeutic form factors to these specific patient populations.

Nonetheless, previous studies have identified several risk factors for poor adherence. In the following sections, we highlight three major factors that contribute to medication adherence. In each section, we discuss the major constraints and considerations associated with each of these factors.

### Route of administration

Self-administration of medication is possible with oral, rectal, and injectable dosage forms. SC injections are the most common method of delivering IBD biologics and are currently the only commercially-available self-administrable formats available. Injectable biologics typically come in either single-dose, single-use prefilled syringes or injection pens. However, lack of confidence in injections and fear of needles are major barriers; indeed, these factors cause many patients to prefer IV delivery over self-administered SC injections, despite the additional hassle of needing to go to the hospital.^22^

In general, most patients prefer oral dosage forms. A study of 298 patients in 2021 found an overwhelming preference for tablets (94%) over granules, IV infusions, and SC injections.^23^ However, oral delivery of biologics is challenging because of the various physiological barriers along the gastrointestinal (GI) tract that reduce bioavailability, including pH gradients across different GI sections, degradative enzymes in the stomach and the intestines, variations in transit time, and the microbial enzymes in the microbiota.^6^ Because of reduced bioavailability, formulating biologic drugs for oral delivery currently requires a larger amount of active pharmaceutical ingredient (API), which can increase production costs.^24^ More studies are needed to ascertain if patient preference actually correlates to improved adherence. Additionally, because of the challenges of creating orally-delivered IBD biologics, existing IBD treatments conflate oral small molecule drugs with SC biologic drugs; in these cases, it is unclear if patient preferences are driven by route of administration or API type.^25^ More research is also needed to understand how preferences vary depending on demographic factors such as patient age, disease stage, and previous medication history.^23^

As an alternative to oral delivery, rectally-administered drugs are advantageous with respect to efficacy because they avoid first-pass metabolism and can be a direct way to access the site of inflammation, particularly for UC treatment.^26^ However, widespread acceptance of rectal dosage forms is hindered by cultural and social stigma.^27^ For example, one study found that intentional non-adherence to rectal mesalamine for UC was common, with 65% of nonadherent patients citing the mode of administration as the primary reason.^28^

### Frequency of administration

For any of the aforementioned routes of delivery, frequency of administration is strongly predictive of adherence.^19^ Administration frequency impacts patient preferences for self-administration as well as behavioral factors like forgetfulness.^29^ For example, patient preference for SC injections over IV infusions is highly dependent on the dosing interval: a 2023 survey reported that 49% of participants preferred injections every 2 weeks compared to an IV infusion every 8 weeks, but that the percent of patients who preferred injections could be significantly increased if the dosing interval was extended to once every 8 weeks as well.^22^ Additionally, despite patient preferences for oral dosage forms, suboptimal adherence persists for oral administration in addition to SC injections, also primarily because of forgetfulness.^30^, ^31^, ^32^

**Fig. 1:**
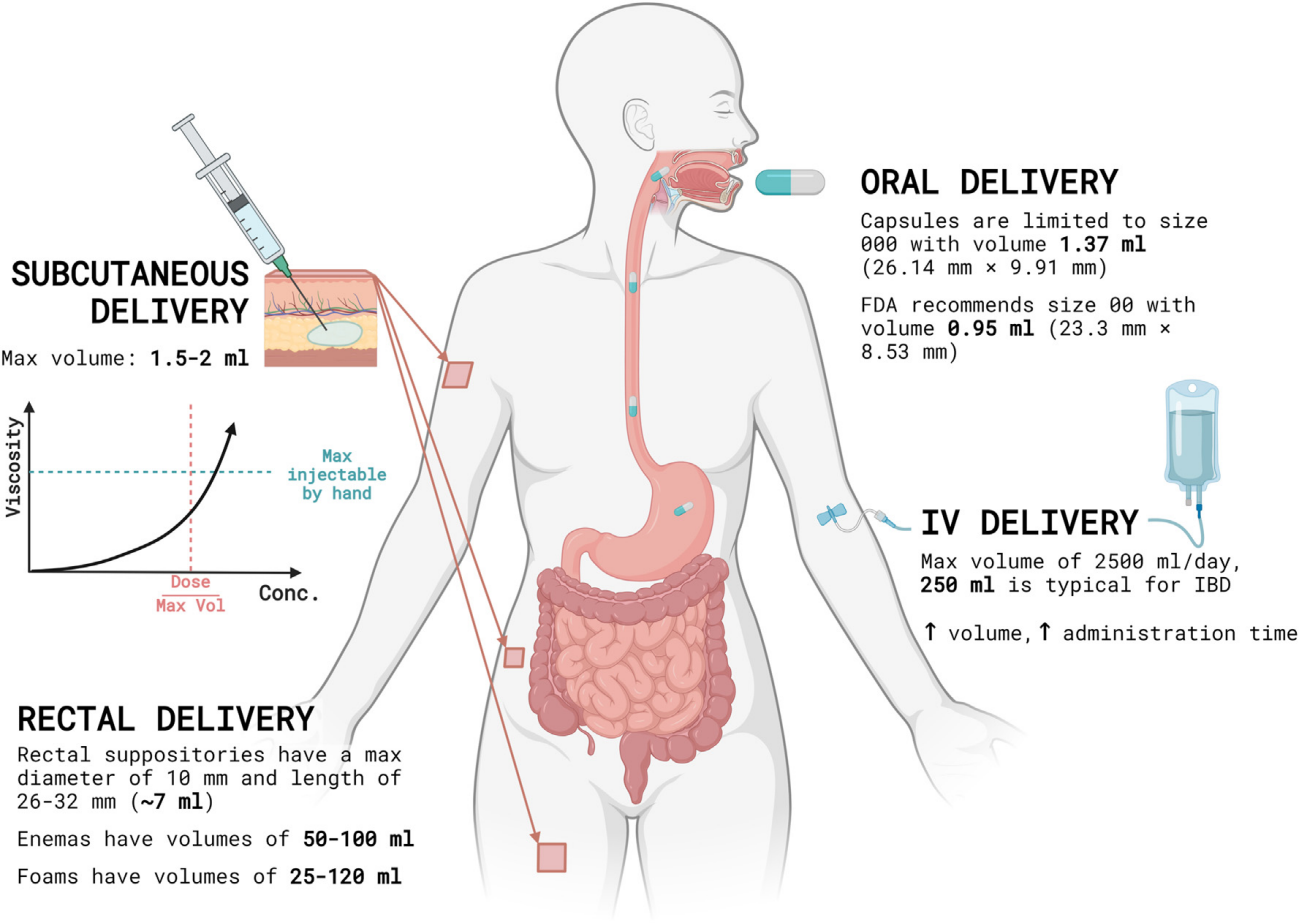
Drug administration routes have general guidelines, but flexibility is often necessary to meet individual needs. Intravenous (IV) infusion volumes vary widely, from small boluses to larger amounts over time, depending on the medication and patient factors. Oral administration, typically through capsules or tablets, can be adjusted based on the patient's ability to swallow, with alternatives like liquids available when needed. Subcutaneous injections are usually given in volumes below the pain threshold, commonly in the abdomen, upper arm, or thigh, though other sites may be used. Rectal delivery offers options including solid suppositories, liquid enemas, and foams, chosen based on the medication and intended effect. These considerations serve as general guidance. Specific medications, patient characteristics, and clinical situations may require different approaches. Always consult current guidelines and product information for precise recommendations.

Frequency of administration largely depends on the amount of bioavailable drug that can be loaded in each dose. The different routes of administration impose different constraints on maximum drug loading (Fig. 1). In the oral and rectal routes, total drug is primarily limited by orifice dimensions along the GI tract,^33^ though degradation of the API also must be accounted for, especially for oral therapies. The maximum size of solid capsules that is generally acceptable for oral administration is a 000 capsule with a total volume of 1.37 ml.^6^ Meanwhile, SC injections without the application of hyaluronidases are generally limited by the maximum volume that can be injected into the subcutaneous space before causing discomfort, which is roughly 1.5 ml,^34^ as well as the maximum viscosity, and therefore injection force that can be administered.^35^ Viscosity also increases as needle gauge decreases; for patient comfort, smaller needles are preferred, and the Centers for Disease Control and Prevention (CDC) recommends needle sizes between 23 and 25 gauge for SC injections.^36^

The maximum injection force that can be applied by hand varies by person, but general quantitative guidelines are available to assist designers. A recent paper surveyed 50 individuals and determined that applied forces of less than 12 N were considered “easy” to inject, whereas materials requiring over 64 N of force were completely non-injectable.^37^ More granularity is needed to understand how these constraints may differ for specific patient populations, including children, the elderly, and the disabled. The relationship between injection force and fluid properties depends on the rheological characteristics of the fluid, the injection speed, and the syringe and needle dimensions.^37^ The most-straightforward drug formulations are Newtonian fluids, whose viscosity are constant with shear rate. Drug formulations for Newtonian fluids possessing viscosities on the order of 1–20 cP have been well-tolerated.^38^

### Undesirable side effects

**Fig. 2:**
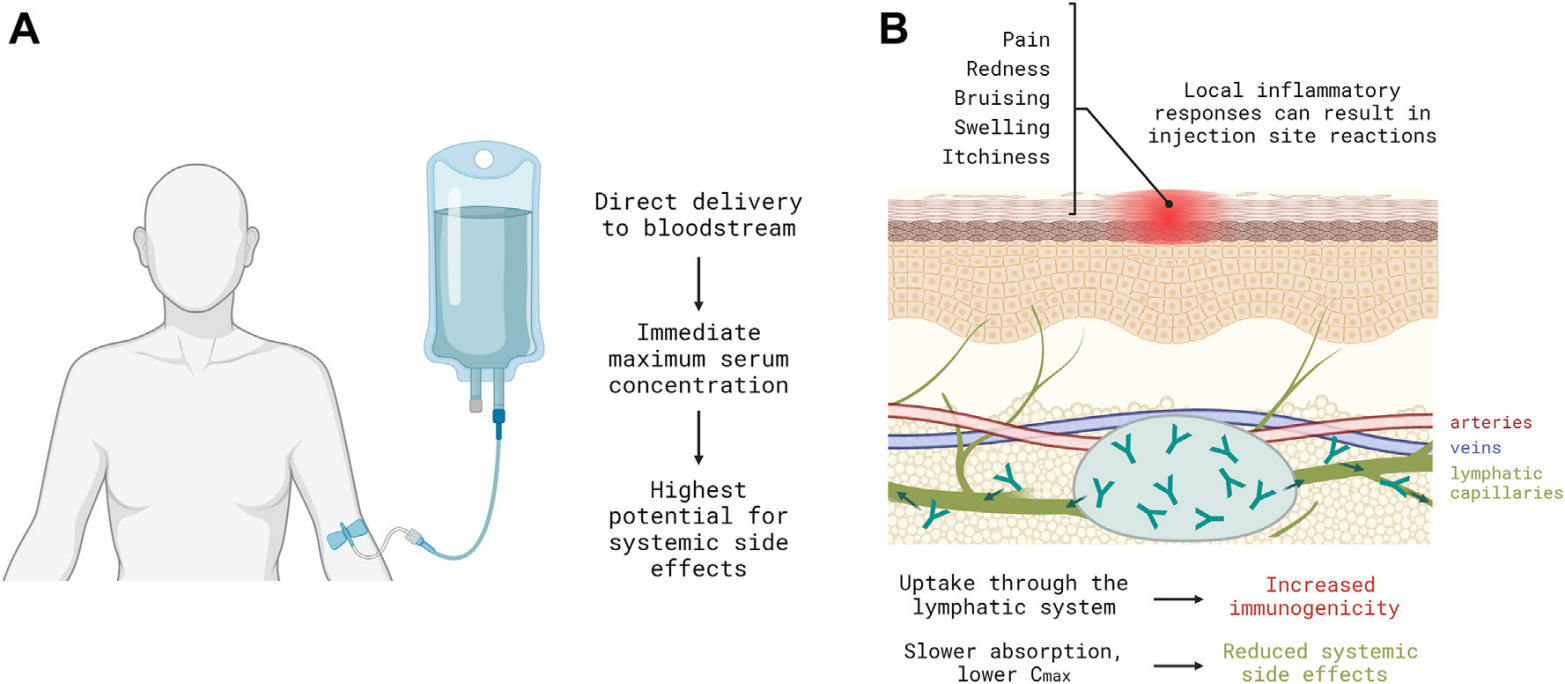
Major sources of undesirable side effects from A) intravenous (IV) infusion and B) subcutaneous (SC) injections.

Finally, side effects from IBD biologics can cause pain, discomfort, and more serious complications. These are important to address, as studies have shown that perception of pain is highly predictive of decreased medication adherence.^39^ For SC injections, factors that impact perception of pain at the injection site include the act of administration (needle size and injection speed), the pharmaceutical product itself (volume, osmolality, and excipients), and allergic reactions associated with drugs and patients’ susceptibility.^40^^,^^41^ Compared to IV infusions, SC administration of biotherapeutics may also be more immunogenic due to the entailment of two waves of antigen presentation by both migratory skin-resident and lymph node-resident dendritic cells, especially with anti-TNF therapies,^42^^,^^43^ although this may not be universally valid—for example, anti-integrin therapies^44^ and anti-IL-12 and anti-IL23^45^ are associated with low immunogenicity. On the other hand, IV infusions directly reach the bloodstream,^46^ usually resulting in immediate maximum serum concentrations (Fig. 2A), while SC-injected therapeutics are characterized by slow absorption from the SC matrix with maximal concentration levels below those achieved with IV dosing.^35^ Following SC injection, biotherapeutics greater than 20 kDa reach the circulation system predominantly via the lymphatic system. This increased exposure to the lymphatic system has led to the suggestion that SC administration of biotherapeutics could be more immunogenic than IV dosing^43^; however, some mitigation strategies on SC-related immunogenicity are promising (Fig. 2B).^43^

Side effects from oral and rectal administration of biotherapeutics have been difficult to assess because of the difficulty in their absorption; biotherapeutics are too large to cross the epithelial barrier to enter the bloodstream and stabilizing biotherapeutics from degradation is challenging in the GI tract. Drug delivery systems are currently being developed for oral and rectal administration so that biotherapeutics can be successfully absorbed and localized to disease sites^47^; these innovations will allow researchers to identify the associated side effects and understand the relative impact they have on patient preferences and adherence.

Overall, sub-optimal route of administration, frequency of administration, and side effects can present notable barriers to medication adherence. Regardless of how powerful a biotherapeutic may be, poor adherence renders therapeutics functionally ineffective. These barriers are inter-related challenges, and must be considered together when designing potential solutions. In the next section, we describe key technological advances and behavior-modifying interventions that promise to address these barriers and create next-generation therapies that not only work well, but that are also used as prescribed.

## Potential solutions to address the multi-factorial adherence challenge

This section highlights approaches that could be helpful for designing solutions to improve medication adherence to IBD biotherapies. Rather than serve as a comprehensive summary, we curate from the literature (“Search strategy and selection criteria”)—including previous review papers on each sub-topic—to identify concepts that are most relevant and impactful for IBD. Ultimately, improving medication adherence will be best achieved with a combination of both near- and long-term approaches: in the near-term, behavior-modifying interventions and technologies compatible with current pharmaceutical products are poised to make the most immediate impacts, whereas in the long-term, several next-generation pharmaceutical technologies are well-positioned to address some of the major barriers to adherence.

### Interventions to modify patient behavior

**Fig. 3:**
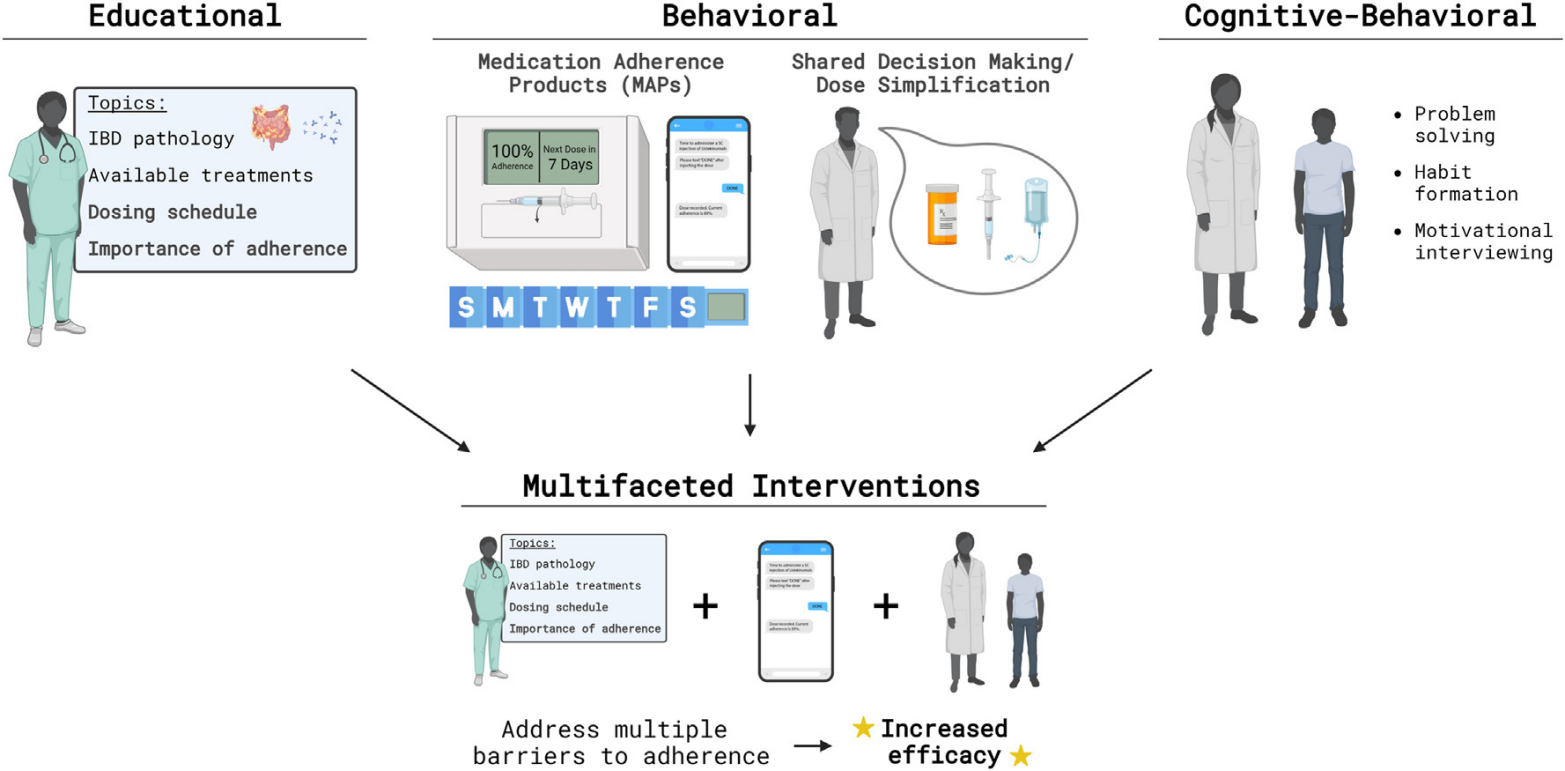
Interventions to modify patient behavior to improve medication adherence.

In the near-term, adherence can be improved using interventions that equip patients with the knowledge, tools, and motivation they need to take existing biotherapeutics as prescribed. These interventions can be classified into educational, behavioral, cognitive behavioral, and multifaceted approaches (Fig. 3).^48^

Educational interventions involve teaching patients about all aspects of IBD, with an emphasis on the details of their treatment plan and the consequences of non-adherence, to help patients make informed decisions that improve their adherence to medication. Pharmacist-led informational counseling and Inflammatory Bowel Diseases Pharmacist Adherence Counseling (IPAC) have been shown to significantly increase medication adherence when assessed 3,^49^ 6,^50^ and 24^51^ months after the intervention; however, an education program consisting of a doctor or nurse-led comprehensive IBD presentation and discussion did not demonstrate a significant improvement at 14 months.^52^ While educational interventions have demonstrated some potential in clinical trials, because the effectiveness of the programming is dependent on a multitude of factors including patient values and willingness to learn, these interventions are often part of multifaceted interventions and are rarely implemented alone.^53^

Behavioral interventions prompt and incentivize medication adherence by providing patients with cues, reminders, and reinforcement. Specifically, medication adherence products (MAPs) and clinical interventions, including shared decision-making between patients and healthcare providers^54^ and dose streamlining,^31^ have been used to improve adherence to IBD medications. MAPs are devices that complement existing IBD biotherapeutics to promote adherence, often with audio-visual reminder, dose organizing, and adherence feedback providing functionalities. Smart MAPs, MAPs with embedded sensing and connectivity, are additionally capable of transmitting usage data to healthcare providers.^55^ One MAP system that sent text reminders to patients for each dose and provided weekly compliance reports resulted in a significant increase in medication adherence at 6 and 12 month time points.^56^ However, a similar Electronic Needle Container (ENC) with reminder and adherence report elements resulted in no significant improvement in medication adherence over 12 months.^57^ This discrepancy reflects the multifactorial nature of the adherence challenge, highlighting the importance of individualizing MAPs selection based on patient needs.

Cognitive-behavioral interventions leverage principles of cognitive-behavioral therapy to help patients overcome negative thoughts that block them from adhering to their medication plan.^58^ This can be accomplished through problem-solving skills training (PSST), motivational interviewing, and habit-formation training.^58^ In a randomized controlled trial, adolescents taking oral medication for IBD were given 2 or 4 sessions of PSST over the phone. Both groups demonstrated significant improvements in adherence and health-related quality of life, with the additional 2 sessions conferring no significant benefit.^59^ Future research should explore the efficacy of PSST in adult populations and of motivational interviewing and habit formation training in all age groups.

A meta-analysis on behavior changing interventions for IBD medication adherence, including both small-molecule pills and injected biologics, was performed in 2022 comprising 17 studies and 7073 patients, and found statistically significant improvements in 12 of the 17 studies.^48^ The largest number of successful studies used multifaceted interventions, combinations of the 3 approaches explored above, highlighting the complex nature of the adherence challenge. Key learnings included the importance of combining educational and behavioral interventions, and the important role of pharmacist counselors and specialized nurses. Notably, a temporal dependence to success was observed: interventions that yielded statistically significant improvements in the near-term often failed to maintain those improvements at a later follow-up date. For example, a study exploring the use of text message reminders by pediatric IBD patients observed statistically significant improvements in adherence 6 months after the start of the intervention, but not after 12 months.^56^

The studies included in the meta-analysis^48^ were assessed across five quality criteria using the Mixed Methods Appraisal Tool (MMAT).^60^ Of the 17 studies included, only 1 study met all 5 quality criteria, and about half of studies met 3 or less, indicating relatively low certainty. Future studies in this area should take care to clearly record and report details regarding administration of interventions and blind outcome assessors to interventions, to reduce bias and increase certainty. Content-wise, future research should study how the effectiveness of behavior modifying interventions depends on the dosage form and drug delivery approach. For instance, educational and cognitive behavioral interventions may be especially impactful to combat patients’ fears about self-administering injections.

### Development of next-generation pharmaceuticals aligned with patient preferences

Rather than aiming to change patient behavior, new technology can improve medication adherence by designing around existing patient behaviors. Technology can improve medication adherence to IBD biologics either by enabling a preferred delivery route (e.g., oral) or by reducing barriers to compliance with self-administrable SC injections. Technologies are classified into either novel drug formulations or novel devices (Table 3). Several comprehensive reviews have been written on these topic areas for biologic delivery in general.^46^ Below, we highlight key technological advances that can be leveraged for the design of next-generation self-administrable IBD biotherapeutics.

**Table 3:** Summary of technological interventions that showcases the potential benefits of each and their relationship to medication adherence.

Technological intervention	Relationship with barrier to adherence		
	Route of administration	Frequency of administration	Undesirable side effects
**Formulations**			
Enabling Oral/Rectal Dosage Forms			
Colon-targeted coatings	Enable oral delivery by stabilizing active ingredient	Increase bioavailable dose by stabilizing active ingredient	Reduce systemic absorption
Inflammation-targeting formulations	Localize to the disease sites to concentrate bioavailable dose	Increase retention of drugs at target site	Reduce off-target effects
Improving SC Injectables			
New biotherapeutics	Improve patient preference for SC injections	Increase potency per dose	Reduce side-effects
Strategies to increase injectable concentration	Improve patient preference for SC injections	Reduce dosing frequency	–
Long-acting formulations	Improve patient preference for SC injections	Reduce dosing frequency	–
**Devices**			
Enabling Oral/Rectal Dosage Forms			
Auto-injecting pills	Enable oral delivery by stabilizing active ingredient	Increase bioavailable dose by stabilizing active ingredient	–
Microneedle robots	Enable oral delivery by stabilizing active ingredient	Increase bioavailable dose by stabilizing active ingredient	–
Jet Injecting Pills	Enable oral delivery by stabilizing active ingredient	Increase bioavailable dose by stabilizing active ingredient	–
Improving SC Injectables			
Auto-injectors and syringe helpers	Improve patient comfort with self-injections	Reduce dosing frequency by enabling larger injection forces	Reduce side effects by programming optimal flow rates and volumes
Needle-free jet injectors	Improve patient comfort for self-administration	Reduce dosing frequency be enabling larger doses to be delivered	Reduce side effects by programming optimal jetting parameters
Microneedle patches	Improve patient comfort for self-administration	–	Avoid injection site pain and discomfort

### New drug formulations

**Fig. 4:**
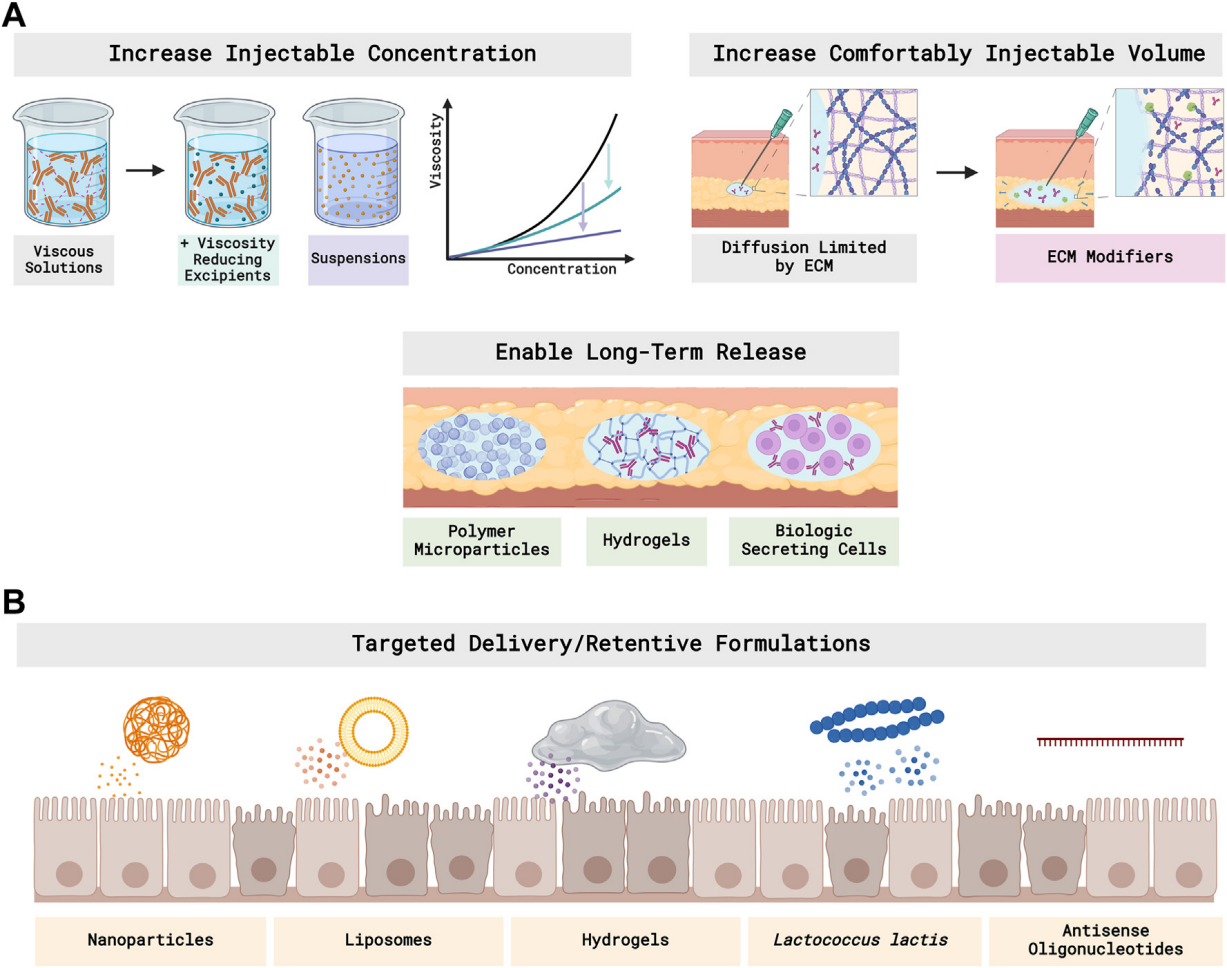
Formulation-based innovations to (A) reduce the frequency of administration and side effects for injectables, and (B) enable and improve oral/rectal dosage forms.

SC injectables can be improved using new formulation approaches (Fig. 4A). For instance, unfavorable side effects can be reduced by developing new biotherapeutics that increase specificity and reduce off-target effects, including IL-23 p19 inhibitor^61^ and IL-6R antagonist.^62^ Injection site discomfort can also be mitigated by reducing total injected volume. Maintaining a constant dose requires higher concentration formulations that still possess adequately low viscosity for manual injection. Similarly, increasing drug concentration for the same volume reduces the required administration frequency, which can also significantly improve patient acceptance for injections.

Conventional biologic formulations are single-phase liquid solutions in which the drug is dissolved in an aqueous carrier fluid. In such solutions, increasing drug concentration causes a steep increase in viscosity due to the presence of multiple intermolecular interactions, including hydrophobic and ionic bonds, thereby limiting the window of concentrations that can be manually injected to a maximum of roughly 100 mg/mL.^63^ Viscosity-modifying excipients like hydrophobic salts extend the window of injectable concentrations by screening intermolecular attractive forces.^64^ The use of excipients will help formulate biologics from IV infusion into SC injection: the successful approval of Envytio (SC vedolizumab) and Zymfentra (SC infliximab) are examples of this conversion.^9^ Although both SC formulations are used for maintenance therapy, which is administered after IV induction therapy, they represent a move one step closer toward self-administered biologics. An alternative approach involves formulating biologics into two-phase suspensions, in which microcrystalline solid forms of the drug are dispersed in a biocompatible non-aqueous solvent such as benzyl benzoate or ethyl lactate. While suspension viscosity also increases with drug concentration, the rate of increase is dramatically slower than for solutions.^65^ Because of this, injectable formulations of model monoclonal antibodies (mAbs) up to 333 mg/mL through 27-gauge needles have been demonstrated.^66^

Rather than increase concentration to maintain a given volume, the ENHANZE® drug delivery technology from Halozyme temporarily increases the maximum volume that can be comfortably injected into the subcutaneous space by degrading part of the extracellular matrix (ECM) at the injection site.^67^ The recombinant human hyaluronidase PH20 enzyme rHuPH20 in ENHANZE degrades hyaluronan, a component of the ECM that limits bulk fluid flow, enabling SC injections of up to 20 mL.^68^ While not yet explored rigorously for co-delivery of IBD biologics, rHuPH20 is currently approved by the United States Food and Drug Administration (FDA) as an adjuvant to increase adsorption of injected drugs, and clinical trials are underway for co-formulations with mAbs for immuno-oncology applications.^69^

Dosing frequency can be further reduced by enabling controlled release of biotherapeutics. One approach involves encapsulating biotherapeutics in polymeric microparticles to limit drug degradation and tune drug uptake rate. Microparticle-based formulations for some small proteins and biosimilars are commercially available^46^ and microparticles encapsulating mAbs have successfully extended-release time frames in preclinical studies,^70^ but formulations for the biotherapeutics used to treat IBD have not yet been developed. Hydrogels can also be leveraged to extend delivery time frames: hydrogel depots containing mAbs have demonstrated prolonged pharmacokinetics in preclinical studies for treating colorectal cancer,^71^ but require further development prior to commercial availability. Alternatively, extended release can be achieved by subcutaneously implanting biologic-secreting cells. Ongoing research in this area aims to optimize cell encapsulation to maintain cell viability, mitigate host inflammatory responses, and ensure constant production and secretion of biologics.^72^ While this technology is still in pre-clinical stages, successful encapsulation and implantation of mAb-secreting cells may present a long-term solution for the treatment of IBD.

Beyond SC injectables, there is a pressing need to develop IBD biologic formulations that are compatible with oral delivery. The central challenge for orally-administered biologics is their limited bioavailability. To date, there are few oral biologic formulations that have attempted phase I/II clinical trials,^46^ and none are yet commercially available.

Multiple formulation approaches are under development to overcome the challenges associated with biologics delivery via the GI tract (Fig. 4B). Existing strategies include colon-targeting coatings to enable biologics delivery via the oral route by protecting against degradation in the upper GI tract.^73^ Enteric coatings like methacrylic acid copolymers (Eudragit)^74^ are commonly used coating materials to prevent disintegration in the upper GI tract owing to their pH-responsiveness; for example, AVX-470 used enteric capsules as carriers in phase I/II clinical trials.^75^ Another reported Eudragit-coated V565 (anti-TNF-α domain antibody) was encapsulated in hydroxypropyl methylcellulose (HPMC) capsules for phase I clinical trials.^76^

Instead of targeting the entire colon, another strategy uses nanomedicine, which has the potential to achieve inflammation-targeting delivery and retention of biologics via the oral or rectal routes, thereby reducing frequency of administration and off-target effects. Nanoparticles (NPs) targeting the inflamed intestine in IBD have been achieved through size-, charge-, ligand-receptor-, degradation-, and microbiota-mediated interaction.^77^ Among them, orally administered new NP formulations or liposomes loaded with biologics have demonstrated improvement in animal models of IBD.^78^, ^79^, ^80^, ^81^ However, limited cases of biologics-loaded NPs have advanced into clinical trials, and further evaluations on safety and manufacturing quality controls of NPs are needed.

Additionally, enemas or foams via rectal administration are topical drug delivery formats directly to the colon, since the colon is the most commonly affected area in IBD. Foams provide increased drug solubility for small-molecule drugs, and mesalamine foams and budesonide foams are available in clinics as first-line therapy in patients with ulcerative proctosigmoiditis.^26^ Inflammation-targeting enema-based hydrogel formulations have also been developed to localize drugs, including anti-inflammatory dexamethasone^82^ and immunosuppressive tacrolimus,^83^ at the inflamed colon and improve therapeutic efficacy in animal models; similar strategies may be applied to biotherapeutics in the future.

Furthermore, engineered bacteria as vectors for biotherapeutics have been widely explored for orally-administered IBD treatment. Genetically engineered *Lactococcus lactis* (*L. lactis*) provided a robust oral delivery platform in animal models of IBD.^84^ The oral formulation AG011 was evaluated in a phase I clinical trial with encouraging results^85^ and entered II clinical trials in patients with moderate UC in 2009. However, no further results were reported.

Lastly, antisense oligonucleotides (ASOs), which are RNA-based therapeutics designed to bind to messenger RNA, are also being explored. An oral SMAD7 ASO, Mongersen, survived phase I and phase II clinical trials,^86^^,^^87^ however, it did not demonstrate efficacy compared to placebo in active CD in its phase III trial.^88^ Altogether, oral delivery of biotherapeutics remains an unfulfilled task and still requires further studies to improve current strategies.

### New drug delivery devices

**Fig. 5:**
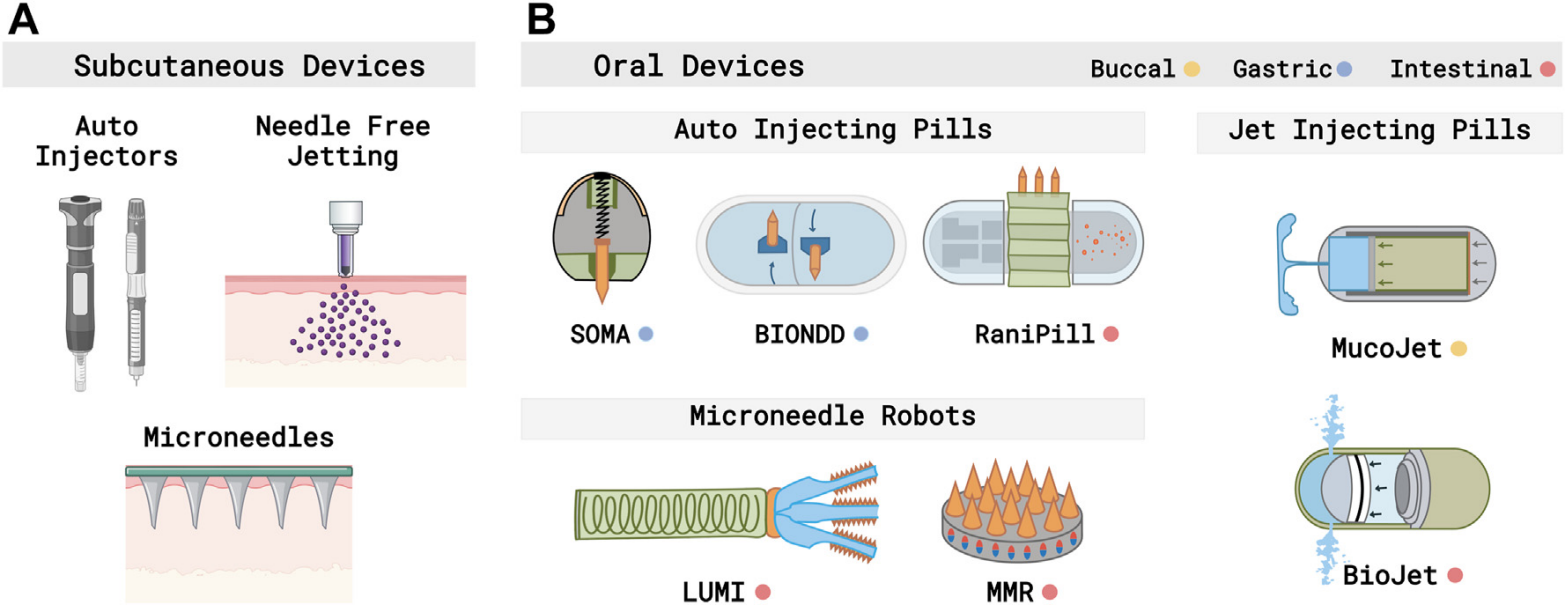
Device-based drug delivery innovations, including (A) to reduce the frequency of administration and discomfort with SC administration and (B) to enable oral dosage of biotherapeutics. (SOMA: Self-Orienting Millimeter-Scale Applicator, LUMI: Luminal Unfolding Microneedle Injector, MMR: Magneto-Responsive Microneedle Robots).

New devices can also address barriers to adherence, either in lieu of or in conjunction with advanced formulations. SC injection devices, including autoinjectors and syringe helpers, can achieve injection forces that exceed human limits, thereby enabling the delivery of higher viscosity formulations (Fig. 5A). Viscosities as high as 1000 cP are deliverable using the Safelia® autoinjector from Nemera, as well as the ArQ®-Bios autoinjector from Oval Medical Technologies.^89^ Higher concentrations of drug may thus be delivered within a given volume, potentially leading to reduced dosing frequency.^41^ By reducing the operational burden on the patient, these devices can also address some of the psychological barriers associated with self-administering injections.

Other devices aim to improve patient comfort with injections by eliminating needles altogether (Fig. 5A). Needle-free jetting injectors have been used for over half a century to administer vaccines and protein drugs via high-speed impact on the skin using either a spring-loaded or gas-powered mechanism. Although injection pain limits their widespread acceptability, new devices address this issue by precisely controlling skin penetration depth to avoid nerve endings, which are more heavily concentrated deeper in the dermis and subcutaneous space.^90^ Jetting systems have an additional advantage of greatly reducing injection times compared to needle-based systems. For delivery of biologic drugs, a tolerability study was performed using the needle-free DosePro jetting system from Zogenix and Batelle. The study found that delivery of 0.5 ml of adalimumab was well-tolerated with the DosePro system, with 81% of participants preferring it over needle injection.^90^ Microneedle patches also reduce pain by avoiding nerve endings and are therefore promising technologies for self-administration. These patches comprise micron-sized drug-loaded conical needles that penetrate the stratum corneum layer of the skin. While microneedle patches for transdermal delivery of mAbs have been demonstrated,^91^ several significant technological challenges remain, including achieving consistent penetration and deposition of drugs across different patients and locations on the body.

Recently, ingestible devices are also being developed to enable oral delivery of biologics by stabilizing the biotherapeutic against degradation and bypassing mucosal and epithelial barriers (Fig. 5B). The first of these devices was the Self-Orienting Milliscale Applicator (SOMA), a robotic capsule that leveraged weight distribution to automatically position a biotherapeutic-loaded microneedle into the gastric mucosa.^92^ Delivery to the intestines was subsequently demonstrated by the RaniPill™, an auto-injecting pill developed by Rani Therapeutics,^93^ and using microneedles actuated via chemical or magnetic stimuli.^94^^,^^95^ As the aforementioned devices rely on the implantation of drug loaded spikes or microneedles into the target tissues, the dosing capacity is strictly restricted by the needle volume. The liquid-SOMA (L-SOMA) device and BIONDD™, from Biograil, address this limitation by auto-injecting liquid formulations into the gastric submucosa through retractable needles, enabling delivery of higher dosages and various formulations.^96^^,^^97^ Biotherapeutics can also be orally administered in the absence of needles through the use of liquid jet delivery. For example, BioJet™, a liquid jetting device by Biora Therapeutics, is in the pre-clinical stage for delivery of adalimumab to the small intestine.^98^

Because a significant portion of the real estate within these devices must be allocated to the mechanical mechanisms that localize and actuate drug delivery, achieving a high loading of drug remains challenging. As such, future research may explore combinations of these devices with the formulation innovations described in the previous section. Additionally, while these devices enable delivery in the GI tract by overcoming barriers to absorption, in each case, the biotherapeutics are taken up systemically rather than delivered directly to the target tissue. Future devices may aim to reduce systemic side effects by integrating tissue targeting strategies alongside delivery mechanisms that overcome the first-pass metabolism.

While technological advancements to existing biotherapeutics have the potential to aid in overcoming multiple barriers to adherence, currently, many of these solutions remain largely theoretical. Therefore, improving medication adherence among IBD patients would benefit from a multidisciplinary approach—one that not only engages scientists and engineers, but also medical professionals in a wide range of roles.

## Conclusions and outlook

**Fig. 6:**
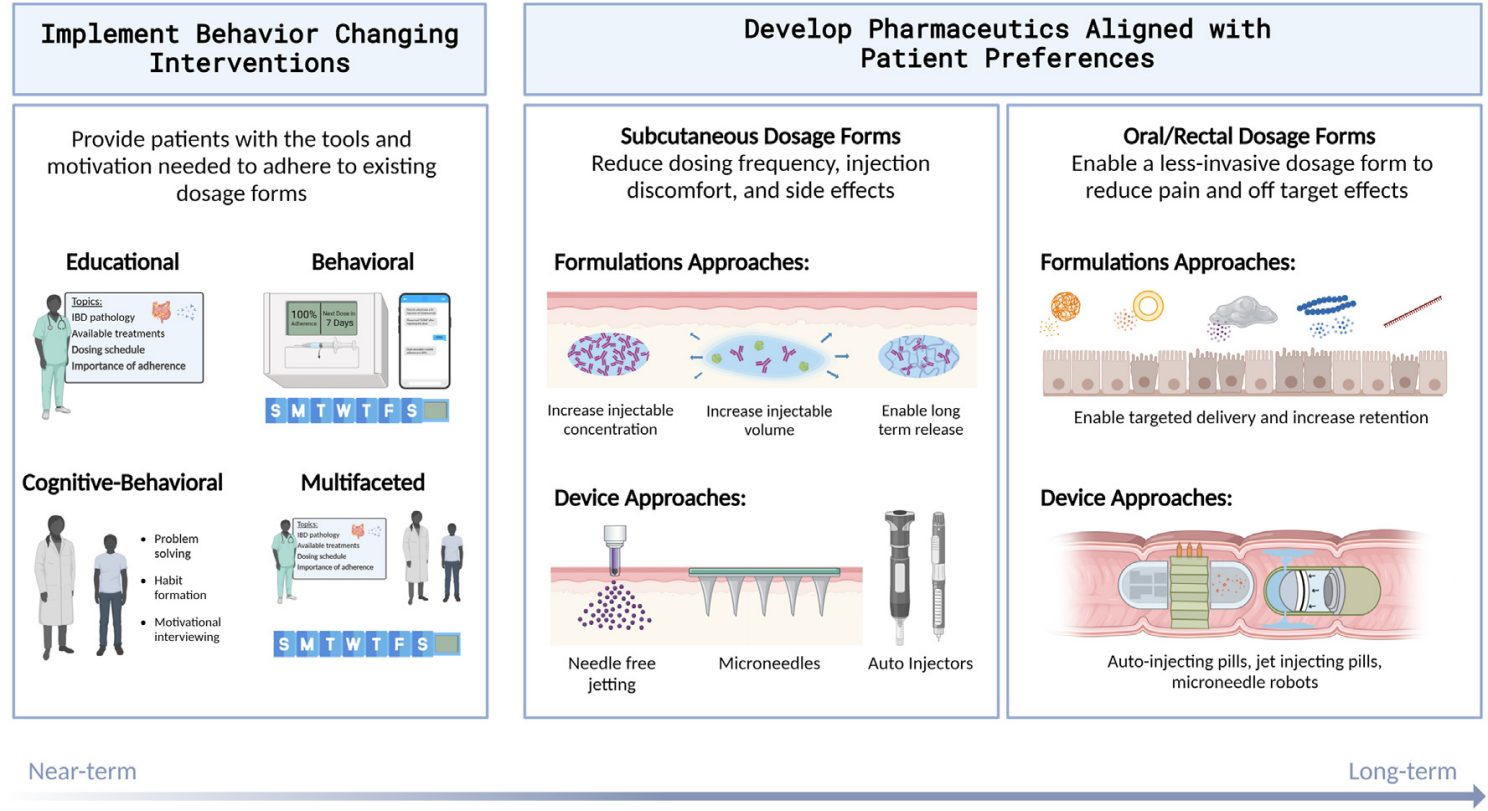
Overview of proposed strategies to increase adherence to biotherapeutics for inflammatory bowel disease (IBD) treatment. In the near term, behavior modifying interventions present a feasible approach to improving adherence to existing biotherapeutics; however, in the long-term, optimal adherence could be best achieved using behavior-modifying and technological approaches in tandem, to comprehensively address patient needs.

Considering the promising efficacy of IBD biotherapeutics, it is critically important to consider how therapies can be designed to maximize patient adherence. We have presented various behavior-modifying and technological strategies to improve adherence; the ideal solution may ultimately depend on the patient, and may require a combination of multiple approaches (Fig. 6). In the near term, behavior-changing interventions may enable improved adherence to existing biotherapeutics; however, as new pharmaceutics become available, shifting to next-generation biotherapeutics may further improve medication adherence and patients' quality of life. Importantly, behavior-changing interventions and next-generation pharmaceutics address different barriers to adherence, and depending on a patient's unique needs, optimal adherence may be best achieved with both approaches in combination.

Moreover, while this review did not discuss the cost of therapies in-depth, cost is undeniably a major barrier to adherence and should be addressed via multiple lenses, including manufacturing and financing systems. Additionally, regulatory considerations must be addressed if new technologies are to be effectively translated to patients, and regulatory pathways may look different depending on whether the innovation is in the form of a new drug molecule, formulation, or device. Finally, home medicine for IBD biotherapeutics also requires out-of-clinic options for therapeutic dose monitoring, including chronic implantable and wearable sensing tools. Regardless of medication adherence, the need to monitor and adjust dosing still drives patients to the clinic, since IBD therapies often fail to elicit therapeutic responses from patients and secondary loss of response during the maintenance phase of treatment is common. Altogether, a concerted multi-disciplinary effort between researchers, clinicians, and industry professionals is needed to realize the vision for IBD home medicine, which has significant potential to improve patients' quality of life.

## Contributors

VRF and GT conceptualized and reviewed the paper. VRF, SZ, and AP performed literature search, wrote, and edited the paper. AP and BS created the figures. ZK, SW, and AB reviewed and edited the paper.

## Data sharing statement

The data used for this review is published literature that is publicly available.

## Declaration of interests

G.T. declares current or prior funding from Novo Nordisk, Hoffman La Orche, Oracle, Draper Laboratory, MIT Lincoln Laboratory, NIH NIBIB and NCI), Bill and Melinda Gates Foundation, The Leona M. and Harry B. Helmsley Charitable Trust, Karl van Tassel (1925) Career Development Professorship, MIT, the Defense Advanced Research Projects Agency, and the Advanced Research Projects Agency for Health (ARPA-H) as well as employment by the Massachusetts Institute of Technology and Brigham and Women's Hospital. Personal financial interests include equity/stock (Lyndra Therapeutics, Suono Bio, Vivtex, Celero Systems, Syntis Bio, GEM-Biosciences, Absco Therapeutics) and royalties (past and potentially in the future) from licensed and/or optioned intellectual property (Lyndra Therapeutics, Novo Nordisk, Suono Bio, Vivtex, Celero Systems, Syntis Bio, Johns Hopkins, MIT, Mass General Brigham Innovation).
